# DrugDoctor: enhancing drug recommendation in cold-start scenario via visit-level representation learning and training

**DOI:** 10.1093/bib/bbae464

**Published:** 2024-09-23

**Authors:** Yabin Kuang, Minzhu Xie

**Affiliations:** College of Information Science and Engineering, Hunan Normal University, 36 Lushan Road, Yuelu District, Changsha 410081, China; College of Information Science and Engineering, Hunan Normal University, 36 Lushan Road, Yuelu District, Changsha 410081, China; Key Laboratory of Computing and Stochastic Mathematics (Ministry of Education), Changsha 410081, China; College of Mathematics and Statistics, Hunan Normal University, 36 Lushan Road, Yuelu District, Changsha 410081, China

**Keywords:** medication recommendation, electronic health record, recommendation system

## Abstract

Medication recommendation is a crucial application of artificial intelligence in healthcare. Current methodologies mostly depend on patient-level longitudinal representation, which utilizes the entirety of historical electronic health records for making predictions. However, they tend to overlook a few key elements: (1) The need to analyze the impact of past medications on previous conditions. (2) Similarity in patient visits is more common than similarity in the complete medical histories of patients. (3) It is difficult to accurately represent patient-level longitudinal data due to the varying numbers of visits. To our knowledge, current models face difficulties in dealing with initial patient visits (i.e. in cold-start scenarios) which are common in clinical practice. This paper introduces DrugDoctor, an innovative drug recommendation model crafted to emulate the decision-making mechanics of human doctors. Unlike previous methods, DrugDoctor explores the visit-level relationship between prescriptions and diseases while considering the impact of past prescriptions on the patient’s condition to provide more accurate recommendations. We design a plug-and-play block to effectively capture drug substructure-aware disease information and effectiveness-aware medication information, employing cross-attention and multi-head self-attention mechanisms. Furthermore, DrugDoctor adopts a fundamentally new visit-level training strategy, aligning more closely with the practices of doctors. Extensive experiments conducted on the MIMIC-III and MIMIC-IV datasets demonstrate that DrugDoctor outperforms 10 other state-of-the-art methods in terms of Jaccard, F1-score, and PRAUC. Moreover, DrugDoctor exhibits strong robustness in handling patients with varying numbers of visits and effectively tackles “cold-start” issues in medication combination recommendations.

## Introduction

The increasing availability of comprehensive health data, such as electronic health records (EHRs), has paved the way for predictive models in clinical decision-making [1–3]. The recommendation of effective and safe medication combinations plays a crucial role in providing appropriate and effective treatment decisions for patients with multiple diseases. Extensive research efforts have been dedicated to the field of medication recommendation [4–9]. Existing methods can be divided into three types: rule-based methods, instance-based methods, and longitudinal methods.

Rule-based methods use predefined rules designed by medical experts to guide the recommendation process [10, 11]. For instance, Chen *et al*. [11] utilized knowledge patterns to code the clinical guidelines for chronic diseases, which serve as rules for medication recommendation. However, rule-based methods heavily rely on expert knowledge and lack generalization capabilities. Instance-based methods focus primarily on the patient’s current health status. LEAP [12] treats the recommendation as a sequential decision-making process. It leverages attention mechanisms to capture dependencies between medication labels in the current visit, and then automatically determines the appropriate medication combinations. Instance-based methods may suffer from lower accuracy due to the insufficient usage of historical visit data.

To overcome this limitation, longitudinal methods incorporate the patient’s historical health records to obtain a more comprehensive understanding of patients’ conditions. Among them, GAMENet [13], SafeDrug [14], and MoleRec [15] utilize Dual-RNN modules to learn patients’ representations from their historical diagnoses and procedure data. However, they all ignore the value of historically prescribed medication information. COGNet [16] develops a novel copy-or-predict mechanism to decide whether to copy a medicine from previous recommendations or to predict a new one. COGNet demonstrates the relevance of historical medications to current prediction. More recently, SHAPE [17] investigates the relationship within the medical events using a compact intra-visit set encoder. It then employs a soft curriculum learning method and makes predictions based on the patient-level representation learned by an inter-visit longitudinal encoder.

Although previous works have shown promising results, they still face two critical limitations: **(1) Challenges in the cold-start scenario**: These models typically rely on patient-level representation derived from the entire historical diagnosis and procedure information of a specific patient. However, similarities among patients may only be evident in certain individual visits rather than across their complete medical histories. As a result, methods based on patient-level representation may not effectively capture the associations between diseases and drugs at the visit level. It means that they hardly generalize known prescription results at the visit level to similar conditions of other patients. Consequently, their predictive performance and application scenarios are limited, particularly when dealing with new patients. **(2) Insufficient utilization of historical prescription information**: Existing methods often focus on using historical diagnosis and procedure information for historical representation, overlooking the impact of historical prescriptions on the patient’s condition. However, it is necessary to recognize that a patient’s current health condition is commonly related to the last treatments, following the administration of previously prescribed medications.

In clinical practice, the prescription process of doctors for patients typically involves the following steps:

- Initially, the doctor evaluates the patient’s health condition based on the diagnosis and procedure information of the current visit. They also consider similar cases they have encountered before (according to their professional experience) to provide an initial prescription.
- Subsequently, the doctor takes into account the patient’s previous prescriptions and the effectiveness of medications in improving the patient’s condition. By incorporating this information, doctors determine the most suitable prescription or combination of medications for the patient’s current visit.

Motivated by this process, we developed a visit-level model named DrugDoctor to address the aforementioned limitations. DrugDoctor focused on extracting correlation information between diseases and drugs prescribed by doctors at the visit level while also investigating the impact of historical medications on the patient’s condition. Specifically, transformer-based encoders were introduced to explore the intrinsic relationship within different medical information. A plug-and-play block named CA-MHSA was proposed to extract information with specific awareness, which incorporates cross-attention and multi-head self-attention mechanisms. We utilized the medication substructure information extracted by a graph neural network as a query to the CA-MHSA block to explore the associations between diseases and medications in the current visit. Additionally, we employed a recurrent neural network and another CA-MHSA block to determine the contributions of historical medications to the present prediction. Different from previous works, to better align with the real-world workflow of doctors, we trained the model in a visit-by-visit manner. The experiments conducted on several widely used datasets showed that DrugDoctor outperformed 10 state-of-the-art methods, especially in the challenging cold-start scenario.

## Problem formulation

###  

#### 0.1 Electronic health records

**Figure 1:**
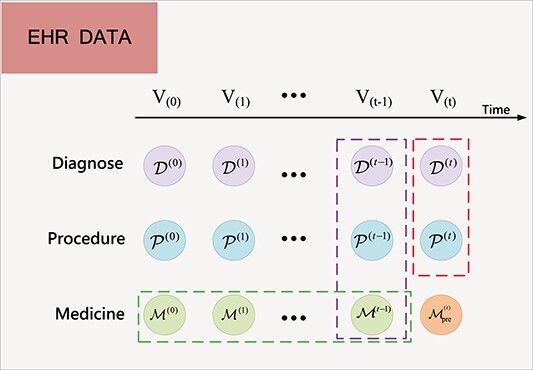
The EHR data of a patient consist of a sequence of visits $V_{(0)},V_{(1)},...,V_{(t)}$; each visit contains a set of medical codes (i.e. the diagnosis code $\mathcal{D}^{(t)}$, the procedure code $\mathcal{P}^{(t)}$, and the medication code $\mathcal{M}^{(t)}$) ; the red box and purple box denote the available information of the current visit and the last visit, respectively,; the green box represents the entire historical prescriptions; $\mathcal{M}_{pre}^{(t)}$ is the predicted medication combinations at current visit.

EHRs are digitally stored and managed personal health information, aiming at providing centralized access and improved decision support. Formally, EHR for patient $i$ can be represented as a time-based sequence $V_{i}=[v_{i}^{(1)},v_{i}^{(2)},\ldots ,v_{i}^{(N_{i})}]$, where $v_{i}^{(t)}$ represents the $t$th visit of patient $i$ and $N_{i}$ is the total number of visits of patient $i$. We omit the patient index $i$ to simplify the notation if there is no confusion. $v^{(t)}$ consists of $\mathcal{D}^{(t)}, \mathcal{P}^{(t)}$, and $\mathcal{M}^{(t)} $, which are, respectively, the set of diagnosis, procedures, and medications that appeared in $t$th visit. It can be further denoted as a concatenation of multi-hot vectors $v_{}^{(t)}=[\mathbf{d}_{}^{(t)},\mathbf{p}_{}^{(t)},\mathbf{m}_{}^{(t)}]$, where $\mathbf{d}_{}^{(t)}\in \{0,1\}^{|\mathcal{D}|},\mathbf{p}_{}^{(t)}\in \{0,1\}^{|\mathcal{P}|},\ and\ \mathbf{m}_{}^{(t)}\in \{0,1\}^{|\mathcal{M}|}$. $\mathcal{D},\mathcal{P},\ and\ \mathcal{M} $ refer to the set of all appeared diagnoses, procedures, and medications, respectively. Figure 1 described the visit-level EHR data.

###  

#### 0.2 Known DDI relation matrix

In real-world clinical practice, it is common to encounter drug–drug interactions (DDIs). DDIs can yield both positive and negative outcomes. However, medication recommendation systems must prioritize patient safety by actively managing the DDI rate, particularly when the potential effects of such interactions remain uncertain. This precautionary approach ensures that the recommended drug combinations are as safe as possible. To achieve this, we utilize a symmetric matrix $\mathbf{D}\in \{0,1\}^{|\mathcal{M}|\times |\mathcal{M}|}$ as prior knowledge to describe the known DDI relation between pairs of drugs. $\mathbf{D}_{ij}=1$ indicates the presence of an interaction between drug $i$ and drug $j$.

###  

#### 0.3 Medication combination recommendation problem

For a patient, given his current diagnosis $\mathcal{D}^{(t)}$, procedures $\mathcal{P}^{(t)}$, historical EHR sequence $[v^{(1)},v^{(2)},\ldots ,v^{(t-1)}]$, and the DDI relation matrix $\mathbf{D}$, medication combination recommendation is to recommend an appropriate combination of medications $\mathcal{\hat{M}}^{(t)}$ for the patient.

## Methods

**Figure 2:**
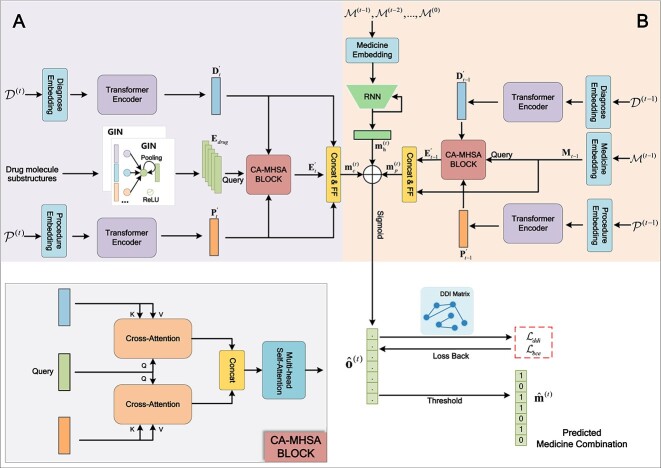
An overview of DrugDoctor; (A) visit-level representation module; it provides the preliminary recommendation $\mathbf{{m}}_{c}^{(t)}$ by exploring the disease–medication associations in the current visit; (B) historical visits learning module; it takes all historical prescriptions and the nearest historical diagnosis and procedure information as input and generates the recommendation based on historical visits, $\mathbf{{m}}_{h}^{(t)}$ and $\mathbf{{m}}_{p}^{(t)}$; in the end, the final prediction results $ {\hat{\mathbf m}}^{(t)}$ are obtained; the details of our proposed CA-MHSA block are also presented in the picture.

As illustrated in Fig. 2, DrugDoctor comprises of three main components: (1) A visit-level representation module that captures the relationship between drugs’ substructures and disease information of the current visit and generates an initial recommendation. (2) A historical visits learning module provides further prediction results by learning all available historical prescriptions and the nearest health condition of the patient after the last administration. (3) A recommendation prediction module calculates the final recommendation results according to the suggestions from the above modules.

###  

#### 0.4 Input representations

We use three learnable embedding tables, $\mathrm{E}_{d}\in \mathbb{R}^{|\mathcal{D}|\times dim},\mathrm{E}_{p}\in \mathbb{R}^{|\mathcal{P}|\times dim}$, and $\mathrm{E}_{m}\in \mathbb{R}^{|\mathcal{M}|\times dim}$ (corresponding to diagnosis, procedure, and medication, respectively), to project corresponding multi-hot vectors into corresponding embedding spaces. Here, $dim$ is the embedding size. Specifically, each row of $\mathrm{E}_{d}$ serves as a unique representation vector corresponding to a specific diagnosis code. For the $t$th visit, when passed through the embedding table $\mathrm{E}_{d}$, the diagnosis set $\mathcal{D}^{(t)}$ is transformed into diagnosis representation $\mathbf{D}_{t}\in \mathbb{R}^{|\mathcal{D}^{(t)}|\times dim}$. Similarly, we obtain the representations of procedure set and medication: $\mathbf{P}_{t}\in \mathbb{R}^{|\mathcal{P}^{(t)}|\times dim}$ and $\mathbf{M}_{t}\in \mathbb{R}^{|\mathcal{M}^{(t)}|\times dim}$, respectively.

###  

#### 0.5 Visit-level representation module

The professional knowledge of doctors is fundamental for prescribing medication to patients. Likewise, in the context of a computational model, it is essential for the model to effectively learn the relationship between diseases and medications. Here, we extract the intrinsic representation of disease information by analyzing the diagnosis and procedure information at the visit level. Usually, there exists a significant correlation between the codes within the diagnosis set (as well as the procedure set).

To capture this intrinsic correlation and obtain more accurate representations, a Transformer-based [18] encoder is introduced to further represent the diagnosis information, which can successfully capture the dependencies between elements in the sequence. To be precise, the encoder consists of two major sub-layers, Multi-head Self-attention layer $\mathrm{MH}(\cdot ,\cdot ,\cdot )$ and Position-wise Feed-Forward Layer $\mathrm{FFN}(\cdot )$. Residual connections are used around each sub-layer, followed by layer normalization. Formally, the Transformer-based encoder can be defined as 


(1) \begin{align*}& \begin{aligned} \begin{aligned} \mathrm{Enc}(\mathbf{X})&=\mathrm{LayerNorm}(\mathbf{H}+\mathrm{FFN}(\mathbf{H}))\\ \text{where H} & =\text{Layer}\mathrm{Norm}(\mathbf{X}+\mathrm{MH}(\mathbf{X},\mathbf{X},\mathbf{X}))\end{aligned}, \end{aligned}\end{align*}

where $\mathbf{X}$ is the input (e.g. $\mathbf{D}_{t}$). $\mathrm{MH}(\cdot ,\cdot ,\cdot )$ is defined as follows: 


(2) \begin{align*}& \begin{aligned} \mathrm{MH}(\mathbf{Q},\mathbf{K},\mathbf{V})=[\mathrm{head}_{1};...;\mathrm{head}_{h}]\mathbf{W}^{O}, \\ \text{head}_{i}=\text{Attention}(\mathbf{QW}_{i}^{Q},\mathbf{KW}_{i}^{K},\mathbf{VW}_{i}^{V}), \end{aligned}\end{align*}

where $\mathbf{W}_{i}^{Q},\mathbf{W}_{i}^{K},\mathbf{W}_{i}^{V}\in \mathbb{R}^{dim\times (dim/h)}\text{ and} \mathbf{W}^{O}\in \mathbb{R}^{dim\times dim}$ are the learnable parameters. $h$ is the number of heads. Attention$(\cdot ,\cdot ,\cdot )$ is calculated as 


(3) \begin{align*}& \begin{aligned} \mathrm{Attention}(\mathbf{Q},\mathbf{K},\mathbf{V})=\mathrm{Softmax}\left(\frac{\mathbf{Q}\mathbf{K}^{\top}}{\sqrt{dim}}\right)\mathbf{V}, \end{aligned}\end{align*}

where $\mathbf{Q}\in \mathbb{R}^{n_{q}\times dim},\mathbf{K}\in \mathbb{R}^{n_{k}\times dim},\mathrm{~and~}\mathbf{V}\in \mathbb{R}^{n_{v}\times dim}$. Another sub-layer $\mathrm{FFN}(\cdot )$ is comprised of two linear layers separated by a ReLU activation. 


(4) \begin{align*}& \begin{aligned} \mathrm{FFN}(\mathbf{H})=\mathrm{ReLU}(\mathbf{HW}_{1}^{F}+\mathbf{b}_{1}^{F})\mathbf{W}_{2}^{F}+\mathbf{b}_{2}^{F}, \end{aligned}\end{align*}

where $\mathbf{W}_{1}^{F}\in \mathbb{R}^{dim\times s},\mathbf{W}_{2}^{F}\in \mathbb{R}^{s\times dim},\mathbf{b}_{1}^{F}\in \mathbb{R}^{s}$, and $\mathbf{b}_{2}^{F}\in \mathbb{R}^{dim}$ are the trainable parameters. According to [18], the inner-layer has dimensionality $s=2048$.

Given the diagnose and procedure representation in $t$th visit, the outputs of corresponding encoders can be formulated as follows: 


(5) \begin{align*}& \begin{aligned} \mathbf{D}_{t}^{\prime}&=\mathrm{Enc}_{\mathbf{d}}(\mathbf{D}_{t})\\ \mathbf{P}_{t}^{\prime}&=\mathrm{Enc}_{\mathbf{p}}(\mathbf{P}_{t}) \end{aligned}\end{align*}

Additionally, the biochemical activity of medications is typically linked to specific molecular substructures, as reported in [19]. Therefore, we believe that analyzing the relationship between the disease and drug substructures can be beneficial for medication recommendation. To obtain drugs’ substructures from their molecules, BRICS [20] method is adopted, which is accessible through the RDKit [21] package.

Since graph isomorphism networks (GIN) [22] have been widely used to learn molecule representation, we introduce a three-layer GIN to encode molecule substructures. Given a molecular substructure graph $\mathcal{G}=\{\mathcal{V},\mathcal{E}\}$, where $\mathcal{V}$ is a set of atoms (i.e. nodes) and $\mathcal{E}$ is a set of chemical bonds (i.e. edges). For atom $v$, GIN encoder represents it by aggregating the features of its neighbors, the layer-wise propagation rule is described as follows: 


(6) \begin{align*}& \begin{aligned} b_{v}^{(k)}=\mathrm{MLP}\Bigg(\left(1+\epsilon^{(k)}\right)\cdot b_{v}^{(k-1)}+\sum_{u\in\mathcal{N}(\nu)}b_{u}^{(k-1)}\Bigg),k=1,2,3, \end{aligned}\end{align*}

where $\operatorname{MLP}(\cdot )$ is a multilayer perceptron, $\epsilon ^{(k)}$ is a learnable parameter, $b_{v}^{(k)}$ is the representation of atom $v$ at the $k$th layer, and $\mathcal{N}(\nu )$ is a set of neighbors of atom $v$. To obtain a global representation of drug substructure, we adopt global mean-pooling on all-atom representations. Finally, we collect the representation of all drug substructures into a table $\mathbf{E}_{drug}$.

Moreover, to enhance the prediction performance, we developed a novel CA-MHSA block based on cross-attention and multi-head self-attention mechanisms to obtain disease information with specific awareness. Precisely, CA-MHSA block is defined as follows: 


(7) \begin{align*}& \begin{aligned} \text{CA-MHSA}(\mathbf{Q},\mathbf{D},\mathbf{P}) = \mathrm{MH}(\mathbf{CA},\mathbf{CA},\mathbf{CA}) \\ \text{where CA} = [\mathrm{Attention}(\mathbf{Q},\mathbf{D},\mathbf{D}), \mathrm{Attention}(\mathbf{Q},\mathbf{P},\mathbf{P})] \end{aligned},\end{align*}

where $\mathbf{Q},\mathbf{D},\ and\ \mathbf{P}$ are the inputs of CA-MHSA block, and $[\cdot ]$ is the concatenate operation. When treating the drug substructures information $\mathbf{E}_{drug}$ as a query, we aim at capturing the drug substructure-aware disease information in the current visit. 


(8) \begin{align*}& \begin{aligned} \mathbf{E}_{t}^{\prime} = \text{CA-MHSA}(\mathbf{E}_{drug},\mathbf{D}_{t}^{\prime},\mathbf{P}_{t}^{\prime}) \end{aligned}\end{align*}

Then, we concatenate $\mathbf{E}_{t}^{\prime }\in \mathbb{R}^{2dim},\mathbf{D}_{t}^{\prime }\in \mathbb{R}^{dim}, \text{ and} \mathbf{P}_{t}^{\prime }\in \mathbb{R}^{dim}$ into a more compact disease representation in $t$th visit. Finally, a feed-forward neural network $\mathrm{FF_{1}}(\cdot ):\mathbb{R}^{4dim}\mapsto \mathbb{R}^{dim}$ is applied to generate a preliminary drug recommendation results, where $dim = |\mathcal{M}|$. 


(9) \begin{align*}& \begin{aligned} \mathbf{{m}}_{c}^{(t)} = \mathrm{FF_{1}}([\mathbf{E}_{t}^{\prime}, \mathbf{D}_{t}^{\prime}, \mathbf{P}_{t}^{\prime}]) \end{aligned}\end{align*}

###  

#### 0.6 Historical visits learning module

For patients with a history of medical visits, DrugDoctor provides additional drug recommendations based on the following two aspects.

**Historical prescription information.** In clinical practice, there is a strong correlation in the drug recommendations for the same patients. For instance, patients with chronic diseases often continue using the same medications over their lifetime. It is observed that a significant portion of the prescribed medicines in most visits are repetitive recommendations [16]. Hence, we use the RNN model to learn historical medication records and provide useful recommendations for current visit. 


(10) \begin{align*}& \begin{aligned} \mathbf{m}_{h}^{(t)}=\mathrm{RNN}\left(\mathbf{M}_{t-1},\ldots,\mathbf{M}_{0}\right) \end{aligned}\end{align*}

For a specific patient, $\mathbf{m}_{h}^{(t)}$ is the predicted result based on all historical medication records of this patient.

**The effect of medication on condition.** In general, a patient’s current state of health is often influenced by the effects of previously prescribed medications on prior conditions. Accordingly, we argue that examining the influence of prior medications on the previous condition is helpful for the current drug predictions. Similarly, we use our proposed CA-MHSA block to fill this task. 


(11) \begin{align*}& \begin{aligned} \mathbf{E}_{t-1}^{\prime} = \text{CA-MHSA}(\mathbf{M}_{t-1},\mathbf{D}_{t-1}^{\prime},\mathbf{P}_{t-1}^{\prime}) \end{aligned}\end{align*}

To be specific, the prior medication information $\mathbf{M}_{t-1}$ is considered as a query to explore its effect on previous diagnose information $\mathbf{D}_{t-1}^{\prime }$ and procedure information $\mathbf{P}_{t-1}^{\prime }$, which are encoded by corresponding Transformer-based encoders. And $\mathbf{E}_{t-1}^{\prime }$ represents the effectiveness-aware medications information. Subsequently, we use a $\mathrm{FF_{2}}(\cdot ):\mathbb{R}^{3dim}\mapsto \mathbb{R}^{dim}$ to predict the current drug combination based on the previous medications and its effectiveness. 


(12) \begin{align*}& \begin{aligned} \mathbf{{m}}_{p}^{(t)} = \mathrm{FF_{2}}([\mathbf{E}_{t-1}^{\prime}, \mathbf{M}_{t-1}]), \end{aligned}\end{align*}

where $\mathbf{E}_{t-1}^{\prime }\in \mathbb{R}^{2dim}\ and\ \mathbf{M}_{t-1}\in \mathbb{R}^{dim}$.

###  

#### 0.7 Recommendation prediction module

After obtaining recommendations from various sources, we naturally merged them together to obtain a more comprehensive prescription recommendation. 


(13) \begin{align*}& \begin{aligned} {\hat{\mathbf o}}^{(t)} = \sigma(\mathbf{{m}}_{c}^{(t)}+\mathbf{{m}}_{h}^{(t)}+\mathbf{{m}}_{p}^{(t)}), \end{aligned}\end{align*}

where $\sigma (\cdot )$ is sigmoid function. Every element of ${\hat{\mathbf o}}^{(t)}$ denotes an appearance probability of the corresponding drug in $t$th prescription. Then, by setting a threshold value $\delta $, we can obtain the recommended drug combination by selecting the entries with values greater than $\delta $, represented as a multi-hot vector ${\hat{\mathbf m}}^{(t)}$.

###  

#### 0.8 Training and inference

**Multiple loss.** The recommendation task can be treated as a multi-label classification task. As a result, we select the binary cross-entropy loss as the loss function for the multi-label task. 


(14) \begin{align*}& \begin{aligned} \mathcal{L}_{bce}=-\sum_{i=1}^{|\mathcal{M}|}\mathbf{m}_{i}^{(t)}log(\hat{\mathbf{o}}_{i}^{(t)})+(1-\mathbf{m}_{i}^{(t)})log(1-\hat{\mathbf{o}}_{i}^{(t)}), \end{aligned}\end{align*}

where subscript represents a drug. Additionally, to control the DDI rate of predicted drug combinations, in line with [14], we defined the DDI loss as 


(15) \begin{align*}& \begin{aligned} \mathcal{L}_{ddi}=\sum_{i=1}^{|\mathcal{M}|}\sum_{j=1}^{|\mathcal{M}|}\mathbf{D}_{ij}\cdot\hat{\mathbf{o}}_{i}^{(t)}\cdot\hat{\mathbf{o}}_{j}^{(t)}, \end{aligned}\end{align*}

In the end, the overall objective function is defined as the weighted combination of $\mathcal{L}_{bce}$ and $\mathcal{L}_{ddi}$, i.e. 


(16) \begin{align*}& \begin{aligned} \mathcal{L}=\mathcal{L}_{bce}+\alpha\mathcal{L}_{ddi}, \end{aligned}\end{align*}

where $\alpha $ is a trade-off parameter to balance the prediction loss and DDI loss.

**Training strategy.** To our knowledge, the training approaches of previous similar medication recommendation models take the whole information (including the entire medical history) of a individual patient as a training unit. Although these approaches could be effective for individuals with extensive medical histories, their performance may be less robust for new patients with limited medical records, making it challenging to extend known prescription outcomes from one patient to another in the cold-start scenario.

The limited generalization capability can be attributed to the fact that similar medical conditions might only present themselves during specific visits instead of persistently across a patient’s overall health history. Each visit serves as a unique snapshot of a patient’s health condition and corresponding treatment needs, and the efficiency of prescribed medications often varies across different visits. Therefore, relying solely on the overall health state of a patient may fail to accurately capture the specific requirements for medications during individual visits.

To overcome this challenge, it is crucial to develop models and approaches that can effectively capture specific features of each visit, taking into consideration the specific medical conditions and treatment requirements at a specific visit. In real-life situations, the order of visits of different patients is random and overlapping. We believe that training the model visit-by-visit is more in line with the practices of doctors.

Based on these insights, we have implemented a fundamentally new training strategy for our model. Given a set of patients, we generate a random sequence of all visits of the patients while preserving the chronological order of each patient’s visits. In essence, the targeted sequence of visits is globally unordered, but the relative order of visits from an identical patient remains unchanged.

DrugDoctor is trained end-to-end with a manner of visit-by-visit, and all the learnable parameters would be optimized. During the inference phase, the model operates following the same pipeline as training. To obtain the recommended drug combination, the threshold value $\delta $ is set to $0.4$ on the output drug representation ${\hat{\mathbf o}}^{(t)}$ in Eqn. (13). By focusing on the visit-level information, it becomes possible to make more accurate and personalized medication recommendations, even for new patients with limited historical data.

###  

#### 0.9 Dataset and metrics

To validate the effectiveness of DrugDoctor, we collected 15 032 hospital admissions records of 6350 patients from MIMIC-III [23] and 23 525 hospital admissions records of 9862 patients from MIMIC-IV [24]. In line with [14], we processed the patients’ EHR data and presented the corresponding statistics in Table 1. Each dataset was typically divided into training, validation, and testing sets using a $ 4:1:1 $ ratio. To achieve visit-level training, each patient set was transformed into targeted visit sequences, which served as the inputs for the model.

**Table 1:** Dataset statistics

	MIMIC-III	MIMIC-IV
# patients	6350	9862
# visits	15 032	23 525
# diagnoses	1958	1999
# procedures	1430	1800
# medicines	112	114
Avg/max # of visits	2.37/29	2.39/66
Avg/max # of diagnoses	10.51/128	8.05/170
Avg/max # of procedures	3.84/50	2.29/41
Avg/max # of medicines	11.65/64	7.67/60
Total # of DDI pairs	448	448
Total # of substructures	491	491

Four common metrics are employed to evaluate the performance: DDI rate, Jaccard Similarity Score (Jaccard), F1-score, and Precision-Recall Area Under Curve (PRAUC). The DDI rate measures the safety of predicted drug combinations and another three metrics are used for evaluating the recommendation efficacy. The detailed definitions of each metric are presented as follows: 


(17) \begin{align*}& \begin{aligned} \begin{aligned} \mathrm{DDI}=\frac{1}{N}\sum_{t=1}^{N}\frac{\sum_{i=1}^{|\hat{\mathcal{M}}^{(t)}|}\sum_{j=i+1}^{|\hat{\mathcal{M}}^{(t)}|}\mathbf{1}\{\mathbf{D}[\hat{\mathcal{M}}_{i}^{(t)},\hat{\mathcal{M}}_{j}^{(t)}]=1\}}{\sum_{i=1}^{|\hat{\mathcal{M}}^{(t)}|}\sum_{j=i+1}^{|\hat{\mathcal{M}}^{(t)}|}1}, \end{aligned} \end{aligned}\end{align*}

where $N$ is the total number of visits of the patient and $\mathbf{D}$ is the known DDI relation matrix. $\hat{\mathcal{M}}_{i}^{(t)}$ denotes the $i$th recommended drug in $t$th visit. $\mathbf{1}\{\cdot \}$ is 1 when $\{\cdot \}$ is true, otherwise is 0. The Jaccard for the patient is calculated as follows: 


(18) \begin{align*}& \begin{aligned} \begin{aligned} \text{Jaccard}=\frac1{N}\sum_{t=1}^{N}\frac{|\mathcal{M}^{(t)}\cap\hat{\mathcal{M}}^{(t)}|}{|\mathcal{M}^{(t)}\cup\hat{\mathcal{M}}^{(t)}|}, \end{aligned} \end{aligned}\end{align*}

where $\mathcal{M}^{(t)}$ is the ground-truth medications set in $t$th visit and $\hat{\mathcal{M}}^{(t)}$ is the predicted result. The F1 of the patient is calculated as follows: 


(19) \begin{align*} & \begin{aligned} \begin{aligned} \text{Precision}_{\text{t}}=\frac{|\mathcal{M}^{(t)}\cap\hat{\mathcal{M}}^{(t)}|}{|\hat{\mathcal{M}}^{(t)}|} \end{aligned} \end{aligned} \end{align*}

(20) \begin{align*} & \begin{aligned} \begin{aligned} \text{Recall}_{\text{t}} =\frac{|\mathcal{M}^{(t)}\cap\hat{\mathcal{M}}^{(t)}|}{|\mathcal{M}^{(t)}|} \end{aligned} \end{aligned} \end{align*}

(21) \begin{align*} & \begin{aligned} \begin{aligned} &\mathrm{F1}=\frac1N\sum_{t=1}^{N}2*\frac{\mathrm{Precision_{t}*Recall_{t}}}{\mathrm{Precision_{t}+Recall_{t}}} \end{aligned} \end{aligned} \end{align*}

The $\mathrm{PRAUC}$ can be calculated as 


(22) \begin{align*}& \begin{aligned} \begin{aligned} \begin{aligned} \mathrm{PRAUC}&=\frac1N\sum_{t=1}^{N}\sum_{k=1}^{|\mathcal{M}|}\mathrm{Precision}(k)_{t}\Delta\operatorname{Recall}(k)_{t} \\ \Delta\operatorname{Recall}(k)_{t}&=\operatorname{Recall}(k)_{t}-\operatorname{Recall}(k-1)_{t}\end{aligned}, \end{aligned} \end{aligned}\end{align*}

where $\mathrm{Precision}(k)_{t}$ and $\Delta \operatorname{Recall}(k)_{t}$ are the precision and the change of recall at cut-off $k$ in ordered retrieval list, respectively.

###  

#### 0.10 Experimental settings

We compare the proposed DrugDoctor with the following 10 baseline methods:

- LR is a standard logistic regression.
- ECC [25] employs multiple SVM classifiers to make predictions.
- LEAP [12] is an LSTM-based generation model. It regards recommendations as a sequential decision-making process based on diagnosis information.
- DMNC [26] utilizes a memory-augmented network with two controllers and a write-protected mechanism to conduct prediction.
- GAMENet [13] integrates the DDI graph through a graph-augmented memory module and utilizes longitudinal patient records for prediction.
- MICRON [27] conducts medication prediction by capturing changes in drugs between different visits using a recurrent residual network.
- SafeDrug [14] introduces molecule structure information to enhance medication recommendation.
- COGNet [16] proposes a copy-or-predict mechanism to generate the medication set.
- MoleRec [15] investigates the relationships between the health condition of patients and molecular sub-structures to improve the prediction.
- SHAPE [17] is a recent approach that proposes a sample adaptive hierarchical medication prediction network to fill the task.

Our model was implemented using PyTorch 1.9.1 with Python 3.8.18 and trained on an NVIDIA GeForce RTX 4090 GPU. The random seed was set to 1023 for reproducibility. It was trained using the Adam optimizer with a learning rate of $5 \times 10^{-4}$ and a batch size of 16. The hyperparameters of the model were selected objectively based on their performance on the validation set. We set the threshold $\delta = 0.4$ and the trade-off parameter $\alpha = 0.5$. The RNN component was implemented using a Gated Recurrent Unit. The testing process was performed according to the previous work COGNet [16]. We randomly sampled $80\%$ of the test data for each evaluation round, repeating this process 10 times. The mean and standard deviation of these 10 rounds were calculated and reported as the final outcome. All the baselines in our study were implemented using the optimized parameters as described in the respective references.

## Results

### Performance comparison

Table 2 presents a comprehensive summary of the prediction performance of all methods on the widely used MIMIC-III dataset. All in all, our proposed DrugDoctor outperforms all baselines with higher Jaccard, F1, and PRAUC scores while maintaining a relatively lower DDI rate. Among the baselines, LR, ECC, and LEAP exhibit poor prediction performance since they only consider patient information within the current visit. On the other hand, the longitudinal-based methods, which consider the patient’s medical history, achieve relatively better performance. MICRON achieves improved performance and a low DDI rate by predicting fewer drugs. SafeDrug and MoleRec both combine molecular representations of drugs and employ specific DDI control strategies to adaptively balance the accuracy and safety of predicted medications.

**Table 2:** Performance comparison on the MIMIC-III dataset; the best results are highlighted in bold

Model	Jaccard $\uparrow $	F1 $\uparrow $	PRAUC $\uparrow $	DDI $\downarrow $	Avg.# of drugs
LR	0.4865 $\pm $ 0.0021	0.6434 $\pm $ 0.0019	0.7509 $\pm $ 0.0018	0.0829 $\pm $ 0.0009	16.1773 $\pm $ 0.0942
ECC	0.4996 $\pm $ 0.0049	0.6569 $\pm $ 0.0044	0.6844 $\pm $ 0.0038	0.0846 $\pm $ 0.0018	18.0722 $\pm $ 0.1914
LEAP(2017)	0.4521 $\pm $ 0.0024	0.6138 $\pm $ 0.0026	0.6549 $\pm $ 0.0033	0.0731 $\pm $ 0.0008	18.7138 $\pm $ 0.0666
DMNC(2018)	0.4864 $\pm $ 0.0025	0.6529 $\pm $ 0.0030	0.7580 $\pm $ 0.0039	0.0842 $\pm $ 0.0011	20.0000 $\pm $ 0.0000
GAMENet(2019)	0.5067 $\pm $ 0.0025	0.6626 $\pm $ 0.0025	0.7631 $\pm $ 0.0030	0.0864 $\pm $ 0.0006	27.2145 $\pm $ 0.1141
MICRON(2021)	0.5100 $\pm $ 0.0033	0.6654 $\pm $ 0.0031	0.7687 $\pm $ 0.0026	0.0641 $\pm $ 0.0007	17.9267 $\pm $ 0.2172
SafeDrug(2021)	0.5213 $\pm $ 0.0030	0.6768 $\pm $ 0.0027	0.7647 $\pm $ 0.0025	**0.0589 $\pm $ 0.0005**	19.9178 $\pm $ 0.1604
COGNet(2022)	0.5252 $\pm $ 0.0011	0.6791 $\pm $ 0.0011	0.7652 $\pm $ 0.0012	0.0876 $\pm $ 0.0005	28.9352 $\pm $ 0.1161
MoleRec(2023)	0.5305 $\pm $ 0.0033	0.6843 $\pm $ 0.0029	0.7736 $\pm $ 0.0027	0.0724 $\pm $ 0.0008	21.0893 $\pm $ 0.1788
SHAPE(2023)	0.5348 $\pm $ 0.0003	0.6885 $\pm $ 0.0003	0.7821 $\pm $ 0.0003	0.0850 $\pm $ 0.0000	20.9008 $\pm $ 0.0777
DrugDoctor	**0.5459 $\pm $ 0.0014**	**0.6975 $\pm $ 0.0014**	**0.7933 $\pm $ 0.0011**	0.0603 $\pm $ 0.0003	20.7996 $\pm $ 0.0647

In particular, COGNet and SHAPE make efforts to learn visit-level knowledge and achieve good results. However, it is worth noting that the implementations of these two models still essentially learn at the patient level, which means their performance may vary depending on the number of patient visits. To demonstrate the effectiveness of visit-level training, we conducted additional comparative experiments of DrugDoctor with COGNet and SHAPE on the MIMIC-IV dataset. The results presented in Table 3 indicate that DrugDoctor possesses enhanced generalization capability compared with COGNet and SHAPE, both of which are representative methods adopting patient-level training.

**Table 3:** Performance comparison on the MIMIC-IV dataset; the best results are highlighted in bold

Model	Jaccard $\uparrow $	F1 $\uparrow $	PRAUC $\uparrow $	DDI $\downarrow $	Avg.# of drugs
COGNet(2022)	0.5084 $\pm $ 0.0019	0.6561 $\pm $ 0.0017	0.7440 $\pm $ 0.0014	0.0927 $\pm $ 0.0004	21.5079 $\pm $ 0.0932
SHAPE(2023)	0.5059 $\pm $ 0.0003	0.6571 $\pm $ 0.0003	0.7428 $\pm $ 0.0002	0.0917 $\pm $ 0.0001	15.4165 $\pm $ 0.0291
DrugDoctor	**0.5103 $\pm $ 0.0016**	**0.6600 $\pm $ 0.0014**	**0.7508 $\pm $ 0.0015**	**0.0705 $\pm $ 0.0007**	14.1290 $\pm $ 0.0819

**Figure 3:**
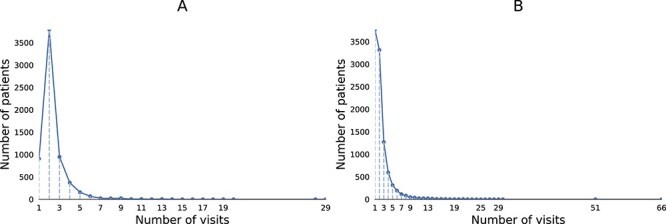
(A) The histogram of hospital visits of a patient in the MIMIC-III dataset; (B) the histogram of hospital visits of a patient in the MIMIC-IV dataset.

**Figure 4:**
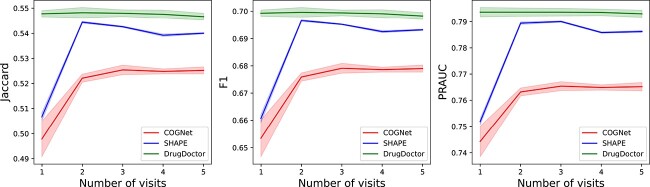
The performances of three models on the patients with specific visits in the MIMIC-III dataset.

**Figure 5:**
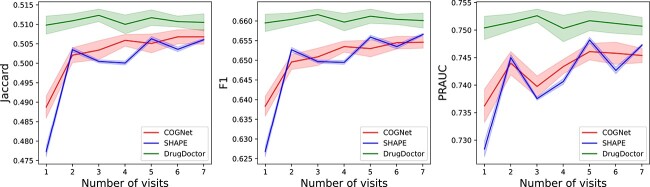
The performance of three models on the patients with specific visits in the MIMIC-IV dataset.

Additionally, to further investigate the robustness of COGNet, SHAPE, and DrugDoctor, we conducted experiments on patients with varying numbers of hospital visits. More specifically, we selected patients with specific numbers of hospital visits, and conducted experiments on the selected patients to assess the models’ performances under different scenarios. Since the majority of patients in the MIMIC-III dataset have fewer than five visits (see Fig. 3 (A)), we focused on the first five visits of patients in the dataset, and tested the models’ performances on the patients with exact $n$ visits for $n = 1... 5$ (Fig. 4). For the the MIMIC-IV dataset, most patients have fewer than seven visits (see Fig. 3 (B)). Therefore we tested the models on the patients in the dataset with exact $n$ visits for $n = 1... 7$, and the experimental results are shown in Fig. 5. Both Fig. 4 and Fig. 5 illustrate that DrugDoctor exhibits remarkable robustness when the number of visits is varying. There are two important observations: firstly, DrugDoctor outperforms COGNet and SHAPE significantly, particularly when dealing with new patients. This indicates that DrugDoctor effectively addresses the cold-start problem. Secondly, all models achieve their highest performance in Fig. 4 when the number of visits is two, which may be attributed to the fact that the proportion of the patients with two visits in the MIMIC-III is much higher than others.

In summary, these findings not only validate the effectiveness of our model, but also highlight its potential in various practical scenarios.

###  

#### 0.11 Ablation study

To verify the effectiveness of each component of DrugDoctor, we design the following ablation models:

- DrugDoctor $w/o\ Block1$: by removing CA-MHSA Block1, the diagnosis information and procedure information are directly combined without the guidance of drug substructure information.
- DrugDoctor $w/o\ Block2$: by removing CA-MHSA Block2, the medication records from previous visits are used without considering the impact of medications on the patient’s condition.
- DrugDoctor $w/o\ DDI$: by removing $\mathcal{L}_{ddi}$ in Eq. 16 from the loss function, the model is trained only by the multi-label classification loss.
- DrugDoctor $w/o\ RNN$: by removing the RNN module, the model ignores the medication records of earlier visits.

Table 4 shows the performance of the different variants of DrugDoctor. As expected, the results of DrugDoctor $w/o\ Block1$ indicate that the relationship between disease and the function of drug substructures is beneficial. And the CA-MHSA $Block1$ could effectively explore the drug substructure-aware disease information in visit-level representation. Similarly, the results of DrugDoctor $w/o\ Block2$ suggest that investigating the influence of prior medications on the previous condition is helpful for the current drug prediction. DrugDoctor $w/o\ RNN$ yields poor results among all ablation models, highlighting the necessity of incorporating historical medication records. Additionally, the results of DrugDoctor $w/o\ DDI$ demonstrate that the combination loss function could effectively balance the accuracy and safety of predicted medication combinations, which is consistent with the fact that the DDI rate typically increases with increasing accuracy. Generally, DrugDoctor outperforms all its variants, indicating the essential role of each component in the model.

**Table 4:** Ablation study of DrugDoctor on the MIMIC-III dataset

Model	Jaccard $\uparrow $	F1 $\uparrow $	PRAUC $\uparrow $	DDI $\downarrow $	Avg.# of drugs
DrugDoctor $w/o~Block1$	0.5387 $\pm $ 0.0014	0.6912 $\pm $ 0.0011	0.7880 $\pm $ 0.0012	**0.0554 $\pm $ 0.0003**	20.8298 $\pm $ 0.0834
DrugDoctor $w/o~Block2$	0.5406 $\pm $ 0.0013	0.6928 $\pm $ 0.0011	0.7896 $\pm $ 0.0009	0.0575 $\pm $ 0.0002	20.6812 $\pm $ 0.0516
DrugDoctor $w/o~DDI$	0.5452 $\pm $ 0.0011	0.6970 $\pm $ 0.0010	0.7923 $\pm $ 0.0012	0.0764 $\pm $ 0.0004	21.6770 $\pm $ 0.1010
DrugDoctor $w/o~RNN$	0.5342 $\pm $ 0.0015	0.6872 $\pm $ 0.0013	0.7819 $\pm $ 0.0013	0.0564 $\pm $ 0.0003	20.5375 $\pm $ 0.0862
DrugDoctor	**0.5459 $\pm $ 0.0014**	**0.6975 $\pm $ 0.0014**	**0.7933 $\pm $ 0.0011**	0.0603 $\pm $ 0.0003	20.7996 $\pm $ 0.0647

###  

#### 0.12 Case study

To intuitively illustrate the advantages of DrugDoctor over COGNet and SHAPE, we randomly sampled several example cases and analyze their predicted results. Due to space limitations, diagnostic and procedural data are abbreviated using International Classification of Diseases (ICD) codes, while medications are encoded via the Anatomical Therapeutic Chemical (ATC) classification system.

**Figure 6:**
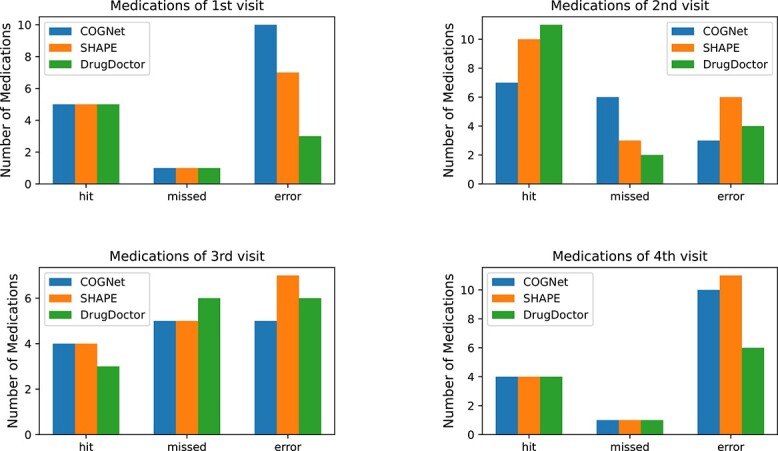
Recommended medications for patient B randomly sampled from the MIMIC-IV dataset, where “hit” and “error” refer to the numbers of medications that are accurately and wrongly recommended, respectively; “missed” denotes the number of medications present in the ground truth prescription that have not been recommended.

Table 5 is about patient A, who was randomly sampled from the patients with two visits in the MIMIC-III test set. In Table 5, “hit” and “error” refer to numbers of medications that are correctly and wrongly recommended, respectively, and “missed” denotes the number of medications present in the ground truth prescription that have not been recommended. As indicated in Table 5, all three models achieved good performance at the second visit of the patient. However, the recommended results for the initial visit clearly demonstrate the distinct advantage of DrugDoctor. Our model obtained more accurate recommendation compared with the other models, with 23 “hit”s and the lowest number of “error”s and “missed”s. To validate the capabilities of DrugDoctor in more complex clinical scenarios, we randomly sampled a patient (called patient B) from the MIMIC-IV test set with four hospital visits. The recommended results for patient B by the models are presented in Fig. 6. DrugDoctor achieved the best performance in the first, second, and fourth visits.

**Table 5:** Recommended medications for patient A with two visits

**First visit**	Code			
Diagnosis: Procedure:	07054,1550,25000,29281,4019,45621,5121,53550,5715,5762,9974,E8780,E9352 0093,3404,3893,4011,4513,5059,5185,5187,9741,9915			
Ground truth:	A02B,A04A,A06A,A07A,A07E,A10A,A12B,A12C,B01A,B05C,C01C,C02C, C02D,C03C,D01A,D07A,D11A,J01C,J01E,J05A,N01A,N02A,N05A,N06A(24 codes)			
Model	Predicted codes	hit	missed	error
COGNet	A01A,A02A,A02B,A04A,A06A,A07A,A07E,A12A,A12B,A12C,B01A,B05C, C01B,C02D,C03C,C07A,D01A,D04A,D07A,D11A,J01C,J01E,J05A,L04A, N01A,N02A,N02B,N05A,N05C(29 codes)	20	4	9
SHAPE	A01A,A02A,A02B,A04A,A05A,A06A,A07A,A07E,A12A,A12B,A12C,B01A, B05C,C01B,C02D,C03C,C07A,D01A,D07A,D11A,J01C,J01D,J01E,J05A, L04A,N01A,N02A,N02B,N05A,N06A,R03A,S01E(32 codes)	21	3	11
DrugDoctor	A01A,A02B,A04A,A06A,A07A,A07E,A10A,A12A,A12B,A12C,B01A,B05C, C01B,C01C,C02D,C03C,C07A,C08C,D01A,D04A,D07A,D11A,H04A,J01C, J01E,J05A,N01A,N02A,N02B,N05A,N05C,N06A(32 codes)	23	1	9
**Second visit**	Code			
Diagnosis: Procedure:	03842,25000,4019,78552,99592,V1007,V427 0331,3893			
Ground truth:	A01A,A02B,A06A,A07A,A07E,A12A,A12B,A12C,B01A,B05C,C01C,D01A, D11A,J01D,J01E,J05A,N02A,N02B,N05B,N06A,R05C (21 codes)			
Model	Predicted codes	hit	missed	error
COGNet	A01A,A02B,A04A,A06A,A07A,A07E,A12B,A12C,B01A,B05C,C01C,C02C,D01A, D07A,D11A,J01C,J01D,J01E,J01M,J05A,N02A,N02B,N05A,N06A (24 codes)	18	3	6
SHAPE	A01A,A02B,A06A,A07A,A07E,A12A,A12B,A12C,B01A,B05C,C01C,C07A, D01A,D07A,D11A,J01C,J01D,J01E,J05A,L04A,N02A,N02B,N06A (23codes)	19	2	4
DrugDoctor	A01A,A02B,A06A,A07A,A07E,A10A,A12A,A12B,A12C,B01A,B05C,C01C,C03C, C07A,D11A,J01C,J01D,J01E,J01M,J05A,N02A,N02B,N05B,N06A,R05C (25 codes)	20	1	5

For patient A, who had only two hospital visits, the recommendation results highlight that DrugDoctor excels in dealing with new patients during their initial visit, successfully generalizing the learned prescription knowledge to these new cases. In the scenario of patient B, who had four visits from a different dataset, DrugDoctor maintained better or competitive performance compared with the other models. Overall, the results of the case studies on these two patients from different datasets further demonstrate the superiority and generalization capabilities of DrugDoctor compared with the representative benchmark methods.

## Discussion and conclusion

Recent advancements in healthcare technologies and the growing availability of patient data have facilitated the development of intelligent medication recommendation systems. This research paper proposes DrugDoctor, a novel drug recommendation model that emulates the decision-making process of human doctors. Unlike previous approaches, we introduce the CA-MHSA block, which explores both drug substructure-aware disease information and effectiveness-aware medication information. Additionally, we achieved fine-grained dataset segmentation, enabling model training at the visit level for the first time. Extensive experiments across various datasets confirmed the superior performance of DrugDoctor compared with other methods and its robustness in handling patients with different numbers of visits.

Importantly, DrugDoctor effectively addresses the prevalent cold-start problem encountered in medication combination recommendations. The effectiveness of DrugDoctor can be attributed to two main reasons. Firstly, both drug substructure-aware disease information and effectiveness-aware medication information extracted by CA-MHSA blocks are beneficial for improving prediction. Secondly, the novel visit-level training strategy enhances the data mining performance of EHR data, as evidenced by the comprehensive comparison with COGNet and SHAPE, which are two representative patient-level training methods.

Despite these advancements, implementing intelligent medication recommendation systems still presents several challenges. Ensuring data privacy and security is paramount, given the sensitive nature of patient information. Furthermore, integrating medication recommendation systems with existing EHR-based platforms requires ensuring data quality and consistency, as EHR data from various sources may differ in format and reliability. Advanced computational infrastructure is also necessary to handle and analyze large volumes of heterogeneous data in real-time. Lastly, enhancing the system’s interpretability and transparency is crucial for gaining healthcare providers’ trust and facilitating effective use in clinical settings.

In conclusion, the development of intelligent medication recommendation systems represents a significant step toward providing personalized and accurate healthcare. However, there are still important areas for further research. Current efforts often focus solely on minimizing DDIs, but it is also essential to recognize that some DDIs can be beneficial, and not all interactions are harmful. Incorporating more comprehensive medication knowledge can help develop rational DDI control strategies that optimize both efficacy and safety. Additionally, recent researches [28, 29] suggested that leveraging structural information from heterogeneous healthcare networks could enhance prediction accuracy. Thus, using heterogeneous graph neural networks to process EHR data might improve recommendation outcomes by capturing rich structured information with fewer data consistency constraints.

**Key Points**

- DrugDoctor is the first drug recommendation model to achieve visit-level representation learning and training, which is more in line with the practices of doctors.
- A plug-and-play CA-MHSA block is proposed to capture the drug substructure-aware disease information and the effectiveness-aware medications information, significantly improving the prediction performance.
- DrugDoctor exhibits a distinct advantage when dealing with the first-time visits of new patients in the cold-start scenario.
- The experimental results on the benchmark dataset validate the effectiveness and robustness of our proposed method, demonstrating the superiority of DrugDoctor over the state-of-the-art baselines.

## Data Availability

The datasets are available online at https://physionet.org/content/mimiciii/1.4/ and https://physionet.org/content/mimiciv/3.0/. The code and results are available at https://github.com/kybinn/DrugDoctor.
